# The Protective Effect of Metformin on Abdominal Aortic Aneurysm: A Systematic Review and Meta-Analysis

**DOI:** 10.3389/fendo.2021.721213

**Published:** 2021-07-28

**Authors:** Zhen Yuan, Zhijian Heng, Yi Lu, Jia Wei, Zhejun Cai

**Affiliations:** ^1^Department of Cardiology, The Second Affiliated Hospital, Zhejiang University School of Medicine, Hangzhou, China; ^2^Department of Oncology, Hospital of Chinese Medicine of Changxing County, Huzhou, China; ^3^Department of Urology, Children’s Hospital, Zhejiang University School of Medicine, Hangzhou, China

**Keywords:** abdominal aortic aneurysm, metformin, expansion rate, aneurysm rupture, inflammation

## Abstract

**Background:**

Type 2 diabetes mellitus (T2DM) patients have a lower risk of abdominal aortic aneurysm (AAA) and its comorbidities, which might be associated with the usage of metformin. The objective of the study was to evaluate the role of metformin in the process of AAA development.

**Method:**

PubMed, Embase and Cochrane Library were searched up to May 15^th^, 2021. We implemented several methods including the risk of bias graph, GRADE system and funnel plot to assess the quality and possible bias of this study. Subgroup analysis and sensitivity analysis were applied to address quality differences and validate the robustness of the final results.

**Result:**

Ten articles were enrolled after screening 151 articles searched from databases. The pooled results showed that, compared with T2DM patients without metformin, metformin prescription was associated with a slower annual growth rate of the aneurysm (mean difference (MD) -0.67 cm [95% confidence interval (CI) -1.20 ~ -0.15 cm]). Besides, metformin exposure was associated with a lower frequency of AAA events (odds ratio (OR) 0.61 [95% CI 0.41-0.92]).

**Conclusion:**

Metformin alleviated both annual expansion rate and aneurysm rupture frequency in AAA patients with T2DM.

**Systematic Review Registration:**

PROSPERO, identifier https://www.crd.york.ac.uk/PROSPERO/display_record.php?RecordID=217859 (CRD42020217859).

## Introduction

Abdominal aortic aneurysm (AAA) is characterized by the permanent dilation of the infrarenal segment of the aorta (1). The prevalence of AAA varied from 3.9 to 7.7% among developed countries in the 1980s and 1990s, and was decreased to 1-2% in recent years (2). In developing countries, however, AAA incidence has been increasing for the past few decades (2). Due to the insidious nature of AAA, AAA is usually discovered accidentally by ultrasonography (3) or presents with catastrophic results like rupture, which accounts for 50-80% of mortality (4). According to the current guidelines, aneurysm repair (including open surgery and endovascular aneurysm repair) is indicated in patients with AAA larger than 5.5 cm in diameter or the onset of symptoms such as abdominal/back/flank pain. Annual screening is suggested for asymptomatic patients with minor AAA (5–7). However, there is no medical therapy available for asymptomatic patients with AAA so far (8).

Type 2 diabetes mellitus (T2DM) is a well-established risk factor of various cardiovascular diseases (CVDs) due to its detrimental effect on microcirculation and median-sized vessels such as coronary arteries (9). Interestingly, researchers found DM was conversely related to the prevalence, incidence, and annual growth rate of AAA (10–12). A multicenter cohort study involving 1.9 million subjects also confirmed T2DM is associated with a lower incidence of AAA (13). There are several different hypotheses on this anomalous phenomenon. Raffort et al. suggested that this resulted from the direct effect of DM on aortic walls, such as mural neo-angiogenesis, intraluminal thrombus formation, inflammation, glycation, extracellular matrix (ECM) remodeling, and vascular smooth muscle homeostasis (10). However, other studies showed that glucose-lowering therapies also had an inhibitory impact on AAA formation (14), which may provide a new medical treatment strategy for asymptomatic patients with AAA.

Metformin, a biguanide-class antidiabetic drug, is the first-line pharmacologic treatment for T2DM, which has been proved to decrease the incidence of cardiovascular events and all-cause mortality in DM patients (14). Besides its role in reducing blood glucose, metformin was also influential in several other areas such as cancer, longevity, and gastrointestinal disorders (15, 16). Recently, metformin was reported to attenuate AAA development and decrease the risk of aneurysm rupture in murine studies and human randomized controlled trials (17–19). Metformin, therefore, may be a potential medical choice for asymptomatic AAA patients. The purpose of this systemic review is to evaluate the effectiveness of metformin in suppressing AAA among patients with T2DM.

## Method

### Literature Search Strategy

Studies were enrolled by exploring electronic databases and scanning reference lists of articles for additional analyses. A comprehensive literature search of PubMed, EMBASE and Cochrane Central Register of Controlled Trials (CENTRAL) was performed to screen qualified articles up to May 2021. The search terms were based on the combination of Medical Subject Heading terms and free words (synonym) and limited English language. The following Medical Subject Heading (MeSH) terms and various text words were used: “Aneurysm, Abdominal Aortic”, “Abdominal Aortic Aneurysm”, “Aortic Aneurysm, Abdominal”, “Metformin”, “Dimethylbiguanidine”, “Glucophage” and “Dimethylguanylguanidine”.

### Eligible Criteria of Reference

Cohort studies, either prospective or retrospective, and randomized controlled trials were included if they met the following criteria: (a) studies included T2DM patients who were prescribed with metformin; (b) reported data of annual aneurysm growth rate, the incidence of rupture of AAA; and (c) published in English. Studies containing duplicate data or overlapping participates were excluded.

### Data Extraction and Quality Assessment

Concerning studies eligible for inclusion, we extracted data to a prespecified table (**Table 1**), which included the first author of the study, year and country of publication, sample size, baseline patient characteristics, primary outcomes, follow-up duration, and endpoint data. Two reviewers independently verified the extracted data.

**Table 1:** Characteristics of the included studies.

Author, year	Region	Study design	Non-metformin	Metformin	Age-y	Outcomes	Study duration	Mean follow-up-y
Sutton, 2020 (20)	USA	Retrospective cohort	43,073	24,361	Non-metformin: 72.27 ± 7.85	Surgery and/or death	2000-2019	NA
					Metformin: 69.65 ± 7.15			
Golledge, 2019 (21)	Australia	Prospective cohort	105	129	Non-metformin: 74.2 ± 7.2	Surgery and/or death as a result of AAA rupture	2.5 ± 3.1y	2.5
					Metformin: 72.4 ± 6.5			
Kristensen, 2020 (22)	Denmark	Case control study	10,375	415	75 (69–80)	AAA rupture	1996-2016	NA
Kristensen,2017 (23)	Denmark	Nested case-control	2,857	1,125	74 (68–79)	AAA rupture	1995-2017	NA
Hsu, 2016 (24)	Taiwan	Nested case-control	5,337	3,599	AAA: 67.5 ± 47.3	Diagnosis of AAA	2000-2013	NA
					non-AAA: 67.5 ± 47.3			
Golledge, 2017 (25)	New Zealand and Australia	Retrospective cohort	132	173	NA	Infrarenal aortic diameters growth (ultrasound in cohort 1; CT in cohorts 2 and 3)	Cohort 1:2002-2015	Cohort 1:3.6
							Cohort 2: 2002-2015	Cohort 2: 2.9
							Cohort 3: 2009-2015	Cohort 3: 1
Unosson, 2020 (26)	Sweden	Retrospective cohort	33	65	Non-metformin: 70.1 ± 6.9	Infrarenal aortic diameters growth determined by ultrasonography	2005-2017	3.2
					Metformin: 68.5 ± 5.4			
Itoga, 2019 (27)	USA	Retrospective cohort	8,392	5,492	69.8 ± 7.8	Infrarenal aortic diameters growth determined by radiographic reports	2003-2013	4.2
Fujimura, 2016 (19)	USA	Retrospective cohort	43	15	72 (56–90)	Infrarenal aortic diameters growth determined by CT	2006-2009	2.6
Wang, 2018 (28)	USA	Retrospective cohort	34	50	Non-metformin: 69.6	CD4^+^ lymphocyte phenotyping, plasma cytokine, antigen and antibody quantification	2015-2017	NA
					Metformin: 69.5			

NA, Not applicable.

After the initial assessment, two authors independently assessed the eligibility of studies identified for potential inclusion. Two authors also completed data extraction and quality assessment independently. The discrepancy was eliminated after discussion. This meta-analysis was conducted in concordance with PRISMA standards of quality for reporting meta-analysis (29).

### Outcome Measures

The primary outcomes of the study were the annual growth rate of AAA and incidence of aneurysm rupture or death.

### Statistical Analysis

The role of metformin in alleviating the annual growth rate of AAA and preventing aneurysm events was investigated. We extracted data information from the eligible trials and use a weighted mean difference with its 95% confidence intervals (CI) to demonstrate the effect of metformin on aneurysm expansion. To compare the incidence of aneurysm events from different studies, we adopted the adjusted odds ratio (OR) with its 95% CI to compute a pooled OR.

The *I* ^2^ index was provided to indicate whether the total variation (in percentage) across studies was attributable to heterogeneity rather than chance. *I* ^2^ >50% indicated substantial heterogeneity. To figure out the origin of heterogeneity, we evaluated covariates that may contribute to heterogeneity with subgroup analysis, based on study scale regarding the number of participants, region, study design, method of imaging studies, and follow-up duration. We also performed a sensitivity analysis to address quality differences and validate the robustness of the final result. We implemented a funnel plot to investigate the publication bias for this study.

Data were analyzed with the Stata MP, version 16.0 software (STATA, College Station, TX) and Review Manager (RevMan) 5.4 (The Nordic Cochrane Centre, The Cochrane Collaboration).

## Results

### Identification of Studies

**Figure 1:**
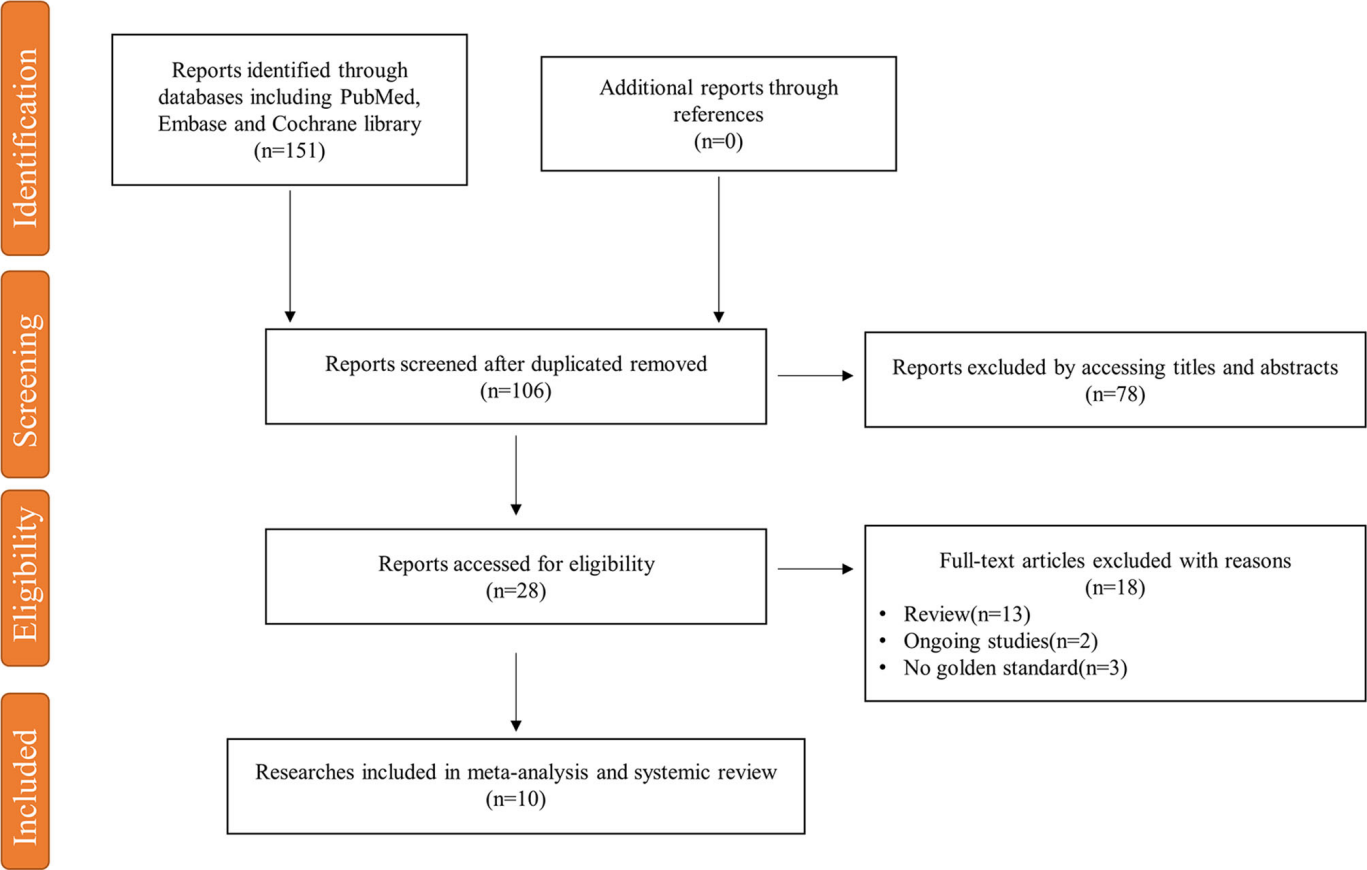
Flowchart of selected studies for inclusion in the meta-analysis.

**Figure 2:**
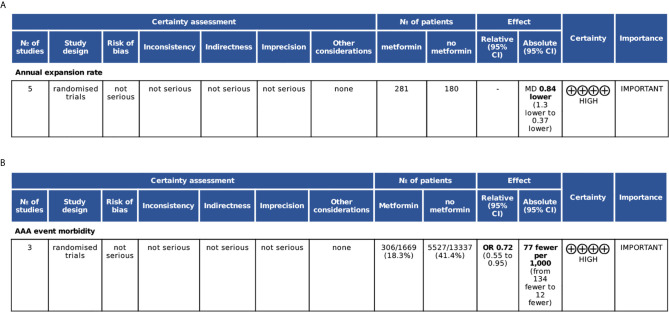
Grading of recommendations Assessment, Development and Evaluation (GRADE) plot. **(A)** Evaluation for annual AAA growth rate analysis. **(B)** Evaluation for incidence of AAA events. CI, confidence interval; MD, mean difference; OR, odds ratio.

The initial literature search identified 151 articles and retained 106 studies after excluding duplicates. After a review of the titles and abstracts, 78 articles were excluded. An additional 18 studies were deleted due to various reasons, including improper article type, unpublished studies, or without golden diagnostic standards. The whole process was shown in the study flow chart (**Figure 1**). There were 10 studies finally included in this study, of which six trials out of 4 studies explored the effect of metformin on attenuating annual aneurysm growth rate, three studies identified its role in decreasing incidence of AAA events, and the others were about other relationships between metformin and AAA, such as anti-inflammation and morbidity. We added the grading of recommendations Assessment, Development and Evaluation (GRADE system) to estimate the evidence quality of analysis on annual AAA growth rate and incidence of events (**Figure 2**).

### Risk of Bias

**Figure 3:**
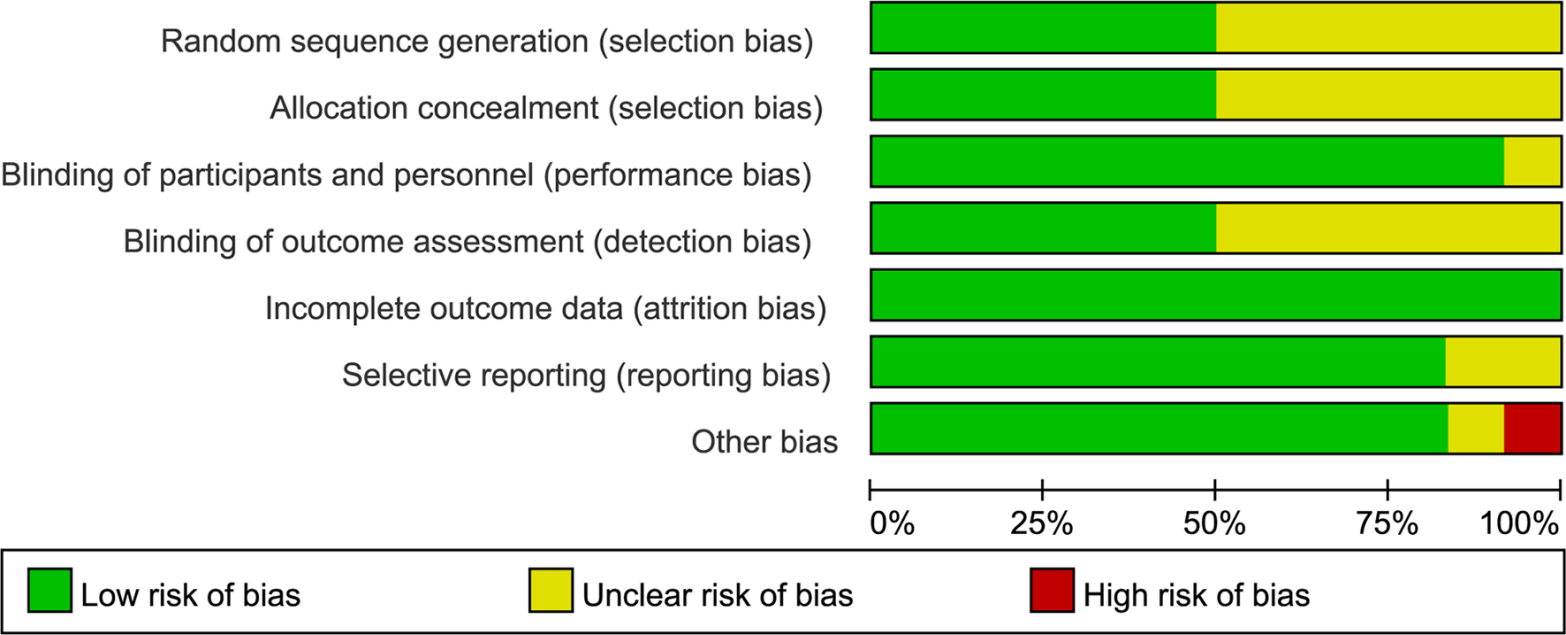
Risk of bias and applicability concerns summary.

**Figure 4:**
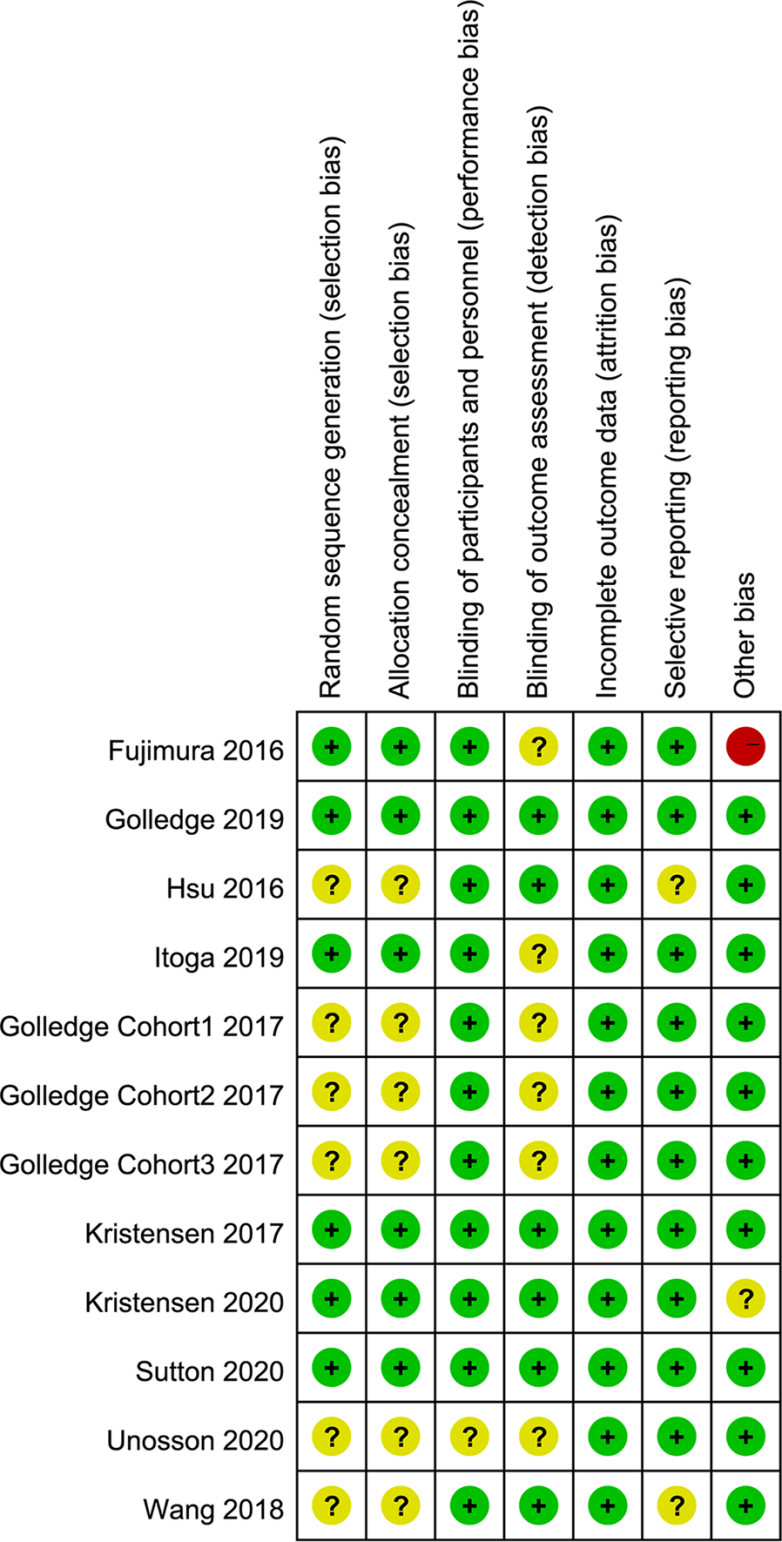
Risk of bias and applicability concerns graph.

We evaluate the quality of enrolled studies with the risk of bias graph and summary (**Figures 3**, **4**), which proved relatively low concern of applicability of these articles. As these diagrams showed, the included studies had a limited risk of bias. One trial was considered to contain a high risk of other bias in the reporting because they indicated their subjects divided into the metformin group were taking or had ever taken metformin (28). Unclear risk of bias was established if there was limited information to exclude associated risk.

### Metformin and AAA Annual Growth Rate

**Figure 5:**
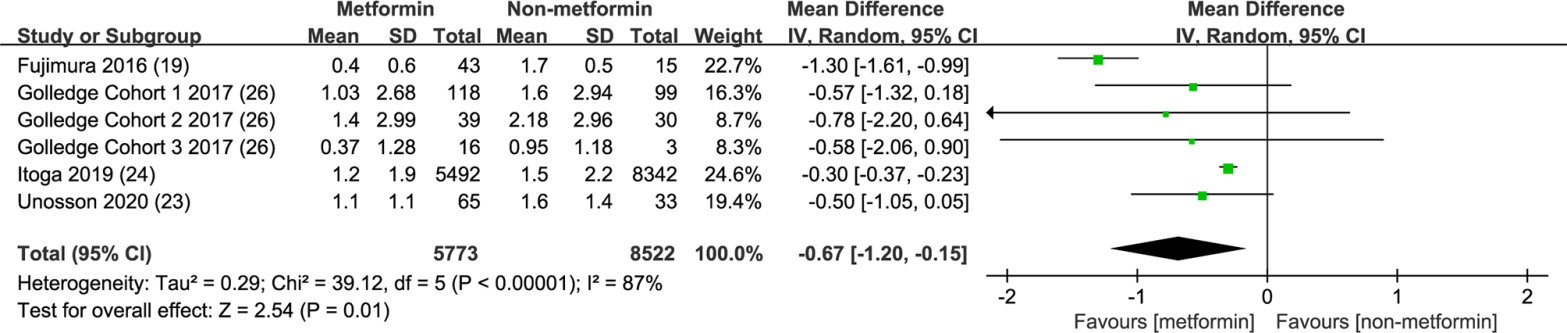
Assessment of the effect of metformin on annual AAA growth rate. Forrest plot of studies assessing the effect of metformin on annual AAA growth rate.

**Figure 6:**
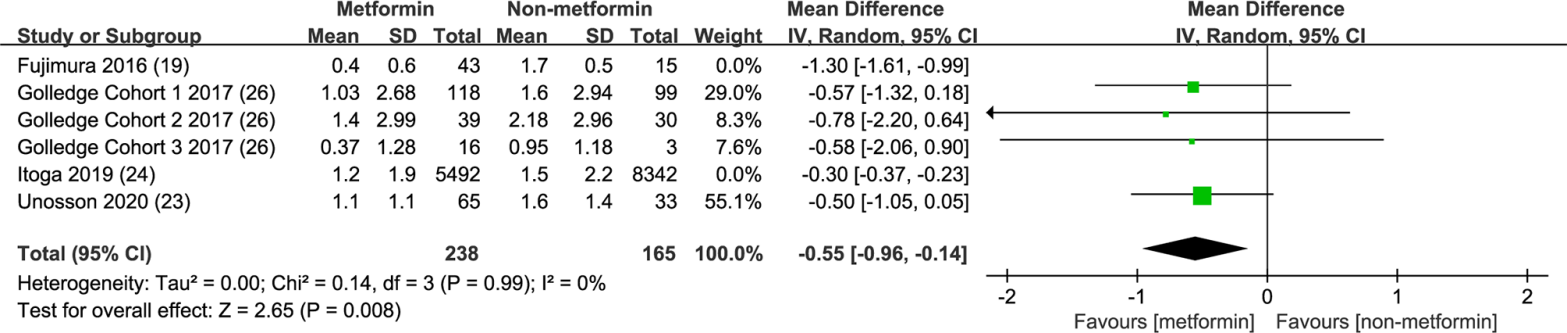
Assessment of the effect of metformin on incidence of AAA. Forest plot of studies assessing the effect of metformin on incidence of AAA events.

We extracted four studies consisting of 6 trials to illustrate the association between metformin exposure and the annual growth rate decline of AAA (19, 25–27) (**Figure 5**). The integrated analysis showed that metformin usage significantly decreased the aneurysm expansion speed, in which the mean difference reached -0.67 cm with a 95% CI from -1.20 to -0.15 cm (p=0.01). However, the *I* ^2^ was 87%. Since the result of Fujimura et al. might be confounded by the inclusion of subjects who had ever taken metformin and the excessively large population of Itoga’s study compared with others, we further pooled data without these two reports. The mean difference turned into -0.35 cm and a 95% CI from -0.42 to -0.29 cm (p<0.00001), with an *I* ^2^ = 0 (**Figure 6**).

### Metformin and Incidence of AAA Events

**Figure 7:**
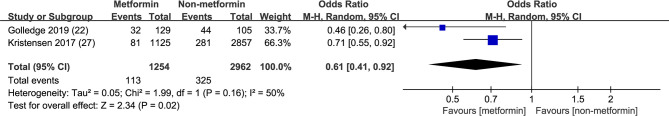
Assessment of the effect of metformin on incidence of AAA after excluding Fujimura 2016. AAA. Forest plot of studies assessing the effect of metformin on incidence of AAA events after excluding Fujimura 2016. *P < 0.05.

Two trials reported the risk of AAA events including death, aneurysm rupture, and need for surgery in T2DM patients (21, 23). The frequency of AAA events is remarkably lower in the metformin group. The pooled adjusted odds ratio of these three studies was 0.61 (95% CI, 0.41-0.92), with *I* ^2^ = 50% (**Figure 7**). Another case-control study demonstrated that metformin did not influence the frequency of AAA events among the general population (22).

### Other Impacts of Metformin on AAA

We also analyzed several other aspects that metformin might influence AAA development from some trials. Hsu et al. conducted a nested case-control analysis using the database extracted from Taiwan’s National Health Insurance Research Database, in which a total of 4468 cases and matched controls were involved (24). This study concluded that metformin prescription was associated with decreased hazard of aneurysm formation. A retrospective cohort study directed by Sutton et al. showed that patients with T2DM had a lower risk of aneurysm repair than subjects without T2DM. Still, they had higher imminence of perioperative mortality (20). However, the mortality was lower in T2DM patients with metformin than patients without T2DM (20). Besides, these patients also had a lower possibility of death in the first ten years after AAA diagnosis (20). Golledge et al. found AAA events might be reduced in T2DM patients with metformin rather than other anti-diabetic therapies compared with those without T2DM (21).

Two articles explored the relationship between metformin and inflammation in AAA patients. Wang et al. collected peripheral blood from patients diagnosed with AAA. They found there was no significant difference of inflammatory cells and cytokines, such as interferon-γ and interleukins, between patients taking metformin or not (28). However, Unosson et al. analyzed samples from 240 patients with AAA and discovered chemokine expression was significantly decreased in those using metformin (26), but the correlation between chemokine level and aneurysm growth rate was not clear. More studies need to be done to prove the association between metformin prescription and AAA expansion and inflammation.

### Publication Bias

**Figure 8:**
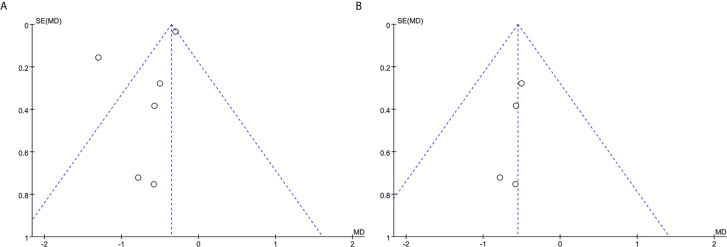
**(A)** Funnel plot of publication bias among studies analyzing AAA growth rate. **(B)** Funnel plot of publication bias after deleting questionable studies.

Publication bias of AAA growth rate was assessed with a funnel plot by observing the symmetry of study distribution (**Figure 8A**). As mentioned above, Itoga 2019 and Fujimura 2016 were excluded with reasons, and the funnel plot for the rest of studies was illustrated in **Figure 8B**. No publication bias was noted in the corrective study.

### Subgroup Analysis and Sensitivity Analysis

To identify if the effect of metformin on AAA annual growth rate varies among different study characteristics, we conducted a subgroup analysis by dividing the included articles into several subgroups depending on subject numbers, study regions, method of imaging studies, and follow-up years (**Table 2**). The result clarified that metformin can downregulate the annual growth rate in diabetic patients independent of study properties. We also implemented sensitivity analysis for these trials, which indicated no significant quality difference among the included studies (**Table 3**).

**Table 2:** Subgroup analysis results.

Subgroup	Population	Study number	Metformin	Non-metformin	Mean difference	Confidendence interval	P value
All combined	Overall	6	5773	8522	-0.67	[-1.20, -0.15]	0.01
Subject number	<100	4	163	81	-0.89	[-1.44, -0.34]	0.001
	≥100	2	5610	8441	-0.3	[-0.37, -0.23]	<0.00001
Region	New Zealand and Australia	3	173	132	-0.61	[-1.22, -0.00]	0.05
	Others	2	5600	8390	-0.7	[-1.41, 0.01]	0.05
Imaging	Ultrasound	2	183	132	-0.52	[-0.97, -0.08]	0.02
	CT	3	98	48	-1.25	[-1.54, -0.95]	<0.00001
	Mixed	1	5492	8342	-0.3	[-0.37, -0.23]	<0.00001
Follow-up	<3 years	3	98	48	-0.52	[-0.97, -0.08]	0.02
	≥3 years	3	5675	8474	-0.31	[-0.37, -0.24]	<0.00001

**Table 3:** Sensitivity analysis results.

Exclued study	Metformin	Non-metformin	Mean difference	Confidendence interval	P value
Fujimura, 2016 (19)	5730	8507	-0.31	[-0.37, -0.24]	<0.00001
Itoga, 2019 (27)	281	180	-0.84	[-1.30, -0.37]	0.0004
Golledge Cohort 1, 2017 (25)	5655	8423	-0.7	[-1.29, -0.10]	0.02
Golledge Cohort 2, 2017 (25)	5734	8492	-0.66	[-1.22, -0.11]	0.02
Golledge Cohort 3, 2017 (25)	5757	8519	-0.68	[-1.24, -0.13]	0.02
Unosson, 2020 (26)	5708	8489	-0.72	[-1.35, -0.08]	0.03

## Discussion

In this paper, we analyzed the role of metformin in AAA development among diabetic patients from distinctive aspects. According to the pooled results, we found that metformin was prone to alleviate the annual growth rate of the aneurysm and reduce the incidence of fatal AAA events, including aneurysm rupture or death. The inverse association between annual growth rate and metformin prescription was observed independent of study populations, regions, imaging methods, or follow-up years. Besides, metformin was related to decreased morbidity of AAA formation in patients with T2DM (24). Prescription of metformin was also accompanied by a lower risk of AAA rupture. Lastly, researchers demonstrated that metformin might down-regulate the inflammatory response such as chemokine production in samples from AAA patients, although it remained to be proved by more studies. These findings indicated that metformin might be a potential medical treatment for asymptomatic patients with AAA.

Metformin is commonly used as an antidiabetic drug that acts in several ways, including suppressing hepatic gluconeogenesis, activating AMP-activated protein kinase, and increasing insulin sensibility in the gut lumen (30, 31). The mechanism that metformin can benefit AAA patients is controversial. Wang et al. implicated that metformin repressed the pathogenesis of aneurysm formation by inhibiting the activation of PI3K/AKT/mTOR/autophagy pathway, which is an important signal pathway regulating cell growth, proliferation, apoptosis, and autophagy of vascular smooth muscle cells (VSMCs) in aortic tissues (18). VSMCs are the main cellular component of aortic walls. Impaired functions of VSMCs might lead to decreased aortic contractility, increased vulnerability to inflammatory cells, and a higher risk of rupture (32, 33). Besides, metformin was shown to be eligible to reduce the formation of atherosclerotic plaques with downregulated serum high-sensitivity C-reactive protein and the activation of NF-κB pathway in the vascular wall, and in the meantime protect vascular endothelial cells (34). Owing to the phenomenon that AAA development is commonly accompanied by aortic atherosclerotic changes within the vascular lumen, metformin may attenuate local inflammatory cell accumulation and hemodynamic changes. Vasamsetti et al. found metformin limited plaque formation and aortic aneurysm in *Apoe* ^−/−^ mice by reducing monocyte infiltration (35). Raffort et al. concluded in their review that metformin prescription is related to changes in the expression of ECM proteins such as alpha1 type IV collagen, alpha2 type XVIII collagen, gamma1, and beta2 laminin (17). These findings might explain why metformin has a protective effect on AAA expansion and rupture.

Studies exploring the mechanism that metformin is beneficial for AAA patients are limited. Although an article claimed that metformin could downregulate serum chemokines in those taking metformin, it demonstrated little correlation between the level of chemokines and aneurysm growth rate (26). The effect of metformin on AAA in patients without T2DM remained unknown. Due to the relatively low prevalence of AAA among diabetic patients, it still lacks solid evidence to show the direct impact of metformin on aortic walls. Some researchers try to identify the effect of metformin on wild-type murine studies. Fujimura et al. found metformin significantly relieved AAA progression with medial elastin and smooth muscle preservation, and suppressed aortic mural macrophage, CD8^+^ T cell infiltration without influencing blood-glucose levels (19). A non-published clinical trial (NCT03507413) showed that metformin could reduce the annual growth rate of AAA in patients without T2DM. Metformin for Abdominal Aortic Aneurysm Growth Inhibition (MAAAGI) Trial is an ongoing multicenter, randomized prospective trial with blinded outcome assessment to evaluate whether metformin reduces AAA growth in non-diabetic patients over five years, of which primary efficacy will be estimated by the difference of AAA diameter compared to baseline (17). There are also several ongoing randomized controlled trials (NCT04224051, NCT03507413, etc.) which may help to identify whether metformin is efficient to prevent AAA development in non-diabetic patients. If the positive associations between metformin using and the prognosis of AAA patients without T2DM can be established, it may become a new medical treatment strategy for these patients.

Several limitations exist in this meta-analysis. AAA development depends on lifestyles, races, and male gender, while this paper did not standardize the included subjects, which may result in confounding bias in part of this meta-analysis. Besides, it should be known that all of the included studies in this article are conducted among subjects with T2DM.

In conclusion, this meta-analysis suggests that metformin can significantly decrease the annual growth rate of AAA compared with patients not taking metformin. Furthermore, metformin is prone to reduce the prevalence of AAA formation and incidence of aneurysm rupture or death from comorbidities. More studies should be encouraged to explore mechanisms of the protective role in AAA patients, which may produce more preventive therapeutic interventions in the future.

## Data Availability Statement

The original contributions presented in the study are included in the article/supplementary material. Further inquiries can be directed to the corresponding authors.

## Author Contributions

ZY and HZ collected data and performed analysis. ZY and ZC wrote the manuscript. ZY, YL, and ZC made revision of the manuscript. All authors contributed to the article and approved the submitted version.

## Funding

This work was supported by funding from the National Natural Science Foundation of China (No. 81970396 to ZC, and No. 81900416 to YL), the Zhejiang Provincial Natural Science Foundation for Distinguished Young Scholars (No. LR20H020002 to ZC), and the Zhejiang Provincial Natural Science Foundation (No. LY19H040012 to JW).

## Conflict of Interest

The authors declare that the research was conducted in the absence of any commercial or financial relationships that could be construed as a potential conflict of interest.

## Publisher’s Note

All claims expressed in this article are solely those of the authors and do not necessarily represent those of their affiliated organizations, or those of the publisher, the editors and the reviewers. Any product that may be evaluated in this article, or claim that may be made by its manufacturer, is not guaranteed or endorsed by the publisher.
